# *Armillaria mellea* Symbiosis Drives Metabolomic and Transcriptomic Changes in *Polyporus umbellatus* Sclerotia

**DOI:** 10.3389/fmicb.2021.792530

**Published:** 2022-02-03

**Authors:** Yong-Mei Xing, Bing Li, Liu Liu, Yang Li, Shu-Xue Yin, Shu-Chao Yin, Juan Chen, Shun-Xing Guo

**Affiliations:** ^1^Key Laboratory of Bioactive Substances and Resource Utilization of Chinese Herbal Medicine, Ministry of Education, Institute of Medicinal Plant Development, Chinese Academy of Medical Sciences & Peking Union Medical College, Beijing, China; ^2^Institute of Fungus Development of Liuba, Qinzheng Zhuling Development Co., Ltd., of Liuba, Hanzhong, China

**Keywords:** metabolomics, UPLC-MS, ergosterol biosynthesis, transcriptomics, *Polyporus umbellatus*, *Armillaria mellea*

## Abstract

Sclerotia, the medicinal part of *Polyporus umbellatus*, play important roles in diuresis and renal protection, with steroids and polysaccharides as the main active ingredients. The sclerotia grow and develop only after symbiosis with *Armillaria* sp. In this study, a systematic metabolomics based on non-targeted UPLC-MS method was carried out between the infected part of the separated cavity wall of the sclerotia (QR) and the uninfected part (the control group, CK) to find and identify differential metabolites. The biosynthetic pathway of characteristic steroids in sclerotia of *P. umbellatus* was deduced and the content of ergosterol, polyporusterone A and B in the QR and CK groups were detected with the High Performance Liquid Chromatography (HPLC). Furthermore, the expression patterns of putative genes associated with steroid biosynthesis pathway were also performed with quantitative real-time PCR. The results showed that a total of 258 metabolites originated from fungi with the fragmentation score more than 45 and high resolution mass were identified, based on UPLC-MS metabolomic analysis, and there were 118 differentially expressed metabolites (DEMs) between both groups. The metabolic pathways indicated that steroids, fatty acid and carbohydrate were active and enriched during *P. umbellatus* sclerotia infected by *A. mellea*. The content of ergosterol, polyporusterone A and B in the QR group increased by 32.2, 75.0, and 20.0%, in comparison to that of the control group. The qRT-PCR analysis showed that series of enzymes including C-8 sterol isomerase (ERG2), sterol C-24 methyltransferase (ERG6) and sterol 22-desaturase (ERG5), which played important roles in the final steps of ergosterol biosynthesis, all presented up-regulated patterns in the QR group in *P. umbellatus*. The comprehensive metabolomic and transcriptomic information will contribute to further study concerning the mechanisms of *P. umbellatus* sclerotial formation infected by *A. mellea* in the future.

## Introduction

*Polyporus umbellatus* (Pers.) Fr. is a traditional medicinal fungus with sclerotia possessing diuretic, antitumor and renal protection acitivity. Polysacchirides and steroids are two main components of the fungus (Zhao et al., 2010; Chen et al., 2017).

In natural conditions, *P. umbellatus* sclerotia grow inside the soil while its edible fruiting bodies germinate from the sclerotia and turn up above the ground (Xing et al., 2011). Due to the habitat destruction, excessive commercial harvesting and lack of effective protection, wild *P. umbellatus* sclerotial resources have become reduced. *Armillaria mellea* is one of the species present in *P. umbellatus* sclerotia and it must form a symbiotic relationship and the sclerotia will grow and develop normally. During the rhizomorphs of *A. mellea* invading *P. umbellatus* sclerotia, the sclerotia form the separated cavities to prevent and restrict *A. mellea* from further entrance to *P. umbellatus*. *A. mellea* then provides nutrition to sclerotia and at the same time, the rhizomorphs branches in the separated cavity and constructs a relatively stable relationship with *P. umbellatus* sclerotia, with the wall of the separated cavity only containing *P. umbelltus* sclerotial tissue while inside the separated cavity, there are *A. mellea* rhizomorphs and sclerotia (Guo and Xu, 1992, 1993; Guo et al., 2011; Xing et al., 2011).

Being one of the most important biological factors and the symbiotic fungus of *P. umbellatus*, *A. mellea* plays essential roles during *P. umbellatus* sclerotial development. In a previous study about transcriptomic analysis between *P. umbellatus* sclerotia with *A. mellea* infection (the QR group) or named the separated cavity part and *P. umbellatus* sclerotia without *A. mellea* infection (the control group), the RNA-Seq data of *P. umbellatus* could be obtained in the NCBI Sequence Read Archive (SRA) database with the accession number of SRP058382 (Liu et al., 2015). Previously, it was found that differentially expressed genes (DEGs) encoding enzymes as Thaumatin-like proteins, WD40 protein, PDR transporter, which were involved in the defense response and transport, were all upregulated in the QR group, in comparison to that of the control group. It had also been well documented that the sugar content containing polysaccharides and glucose in the *A. mellea* infected part of *P. umbellatus* sclerotia was higher than that of the part without *A. mellea* infection (Guo et al., 2002). These results indicated that the differential metabolites present in the QR and the control parts might be different during *A. mellea*’s infection to *P. umbellatus* sclerotia and affect the composition of the metabolites residing in the medicinal fungus. Thus, it is of great importance to conduct related research to uncover the differential metabolites between the different two parts of *P. umbellatus* sclerotia.

Metabolomics, containing non-targeted and targeted methods, is a high-throughput technology and focuses on the small chemical molecules involved in metabolism or metabolic response (Rinschen et al., 2019; Wen et al., 2019; Zhao et al., 2019). As one of the most powerful metabolomic tools, UPLC-MS utilizes an analytical strategy to detect as many metabolites as possible (Dunn et al., 2011). Most of the studies about *P. umbellatus* and *A. mellea* has focused on the taxonomy of these fungi and the effects of the external conditions on *P. umbellatus* sclerotial formation, however, the metabolomic changes of *P. umbellatus* sclerotia due to *A. mellea* infection have not been reported. Therefore, it gives rise to our interest in identifying the different metabolites comprehensively. In this study, a systematic metabolomic study based on non-targeted UPLC-MS was carried out between the separated cavity wall (QR) and the control group of *P. umbellatus* sclerotia after *A. mellea* infection, in order to find characteristic DEMs with pharmaceutical effects which have been in previous studies. In addition, combined with the transcriptomic data, the metabolic pathway was also analyzed. The expression pattern of the candidate DEGs related to the final steps of ergosterol biosynthesis was detected using real-time qRT-PCR method. Furthermore, the content of ergosterol, polyporusterone A and B in the QR and control groups were detected using HPLC. This study will facilitate to provide some new information of metabolites in investigating further studies on symbiotic relationship between *P. umbellatus* sclerotia and *A. mellea*.

## Materials and Methods

### Fungal Growth Conditions

The solid medium was composed of a mixture of wheat bran and sawdust, with the mass ratio being 1:3, after that, sucrose aqueous solution with the mass-to-volume of 1% was added to the culture substrate to make the water content to 70%. Subsequently, the culture medium was subjected to autoclave sterilization at 122°C for 3 h and then after the sterilized medium was cooled, *A. mellea* was transferred from the PDA medium to the solid culture and cultivated in the dark at 25°C. One month later, *A. mellea* was transferred to the stick culture medium, which contains sterilized short sticks (about 250 g) and water with the total volume of 2/3 in plastic bottles and cultivated at 25°C in the dark. After 2 months, *A. mellea* was ready for co-cultivation with *P. umbellatus* sclerotia in the field.

### Co-cultivation of *Polyporus umbellatus* and *Armillaria mellea* in the Field

*Armillaria mellea* and *P. umbellatus* were cultured in Guxian of Shanxi province. Briefly, the co-cultivation experiment was performed in caves with each length × width × height being 40 cm × 40 cm × 30 cm. Three sticks with 30-cm long bearing 4 oblique cuts were placed in parallel and in equal distance in each cave. Then, 250 g of *P. umbellatus* sclerotia was evenly put on both sides of the sticks or beside the location of the cut. *A. mellea* growing in one bottle was put closely to *P. umbellatus* scleroita. Humus soil was filled with the gap and 250 g of thin branches were placed on the surface. Finally, 200 g chestnut leaves were sprinkled on the top. After 1 year and a half, the samples of the QR and the control groups were collected for metabolomic analysis and the content of ergosterol, polyporusterone A and B detection.

### Sample Collection and Pretreatment for Metabolomic Analysis

**FIGURE 1:**
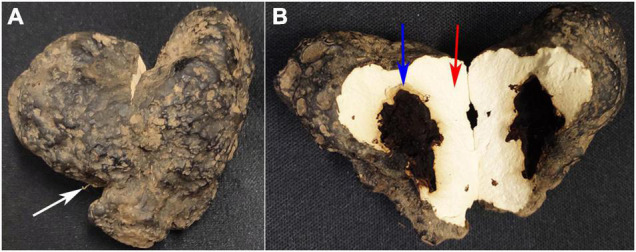
Different partspc collected from *Polyporus umbellatus* sclerotia. **(A)** Represents the whole *P. umbellatus* sclerotium with *A. mellea* rhizomorph (white arrow) invading the sclerotium. **(B)** Is a vertical section of *P. umbellatus* sclerotium. The position marked with the blue arrow represents the wall of the cavities in sclerotia infected by *A. mellea* (the QR part). The part marked with the red arrow stands for the uninfected part of *P. umbellatus* sclerotium without *A. mellea* (the control part).

According to Figure 1, the intact sclerotium was presented in Figure 1A, with *A. mellea* rhizomorph (white arrow) invading *P. umbellatus* sclerotium. The content inside the separated cavity was discarded and the wall of the separated cavities in *P. umbellatus* sclerotia was obtained as the QR group with blue arrow (Figure 1B) and the uninfected part with the red arrow (the control group, Figure 1B) were, respectively, collected. In each group, a 60 mg sample was obtained after *A. mellea* and *P. umbellatus* were co-cultivated for one and a half years, with 7 biological replicates in each group.

The procedure for sample pretreatment was as follow: first, the samples in each group were accurately measured, and then 20 μL L-2-Chlorophenylalanine methanol solution with the concentration of 0.3 mg/mL and 0.6 mL methanol solution with the volume ratio of methanol and water to be 7:3 (V/V) were in turn added. Subsequently, after the samples were put in −20°C for 2 h and subjected to be grinded (60 Hz) for 2 min then ultrasonic extraction was performed for 30 min and again placed them in −20°C for 2 h. Finally, after being centrifuged for 15 min (13000 rpm, 4°C), the 200 μL supernatant was collected into the sample vials for further analysis.

### Metabolomic Tests for *Polyporus umbellatus* Sclerotia

The analytical platform was ultra-performance liquid chromatography combined with time of flight mass spectrometer system (Waters, US). The chromatographic condition was as follows: the chromatographic column (100 mm × 2.1 mm i.d., 1.7 μm; Waters, Milford, MA, United States), the mobile phase A (water containing 0.1% formic acid) and the mobile phase B (acetonitrile containing 0.1% formic acid). The gradient elution program was as follows: 5%∼20% B for 2 min, 20%∼60% B for 8∼12 min, 60%∼80% B for 8∼12 min, 100% B for 2 min, 100% to 5% B for 14∼14.5 min, 5% B for 1 min. The flow velocity was 0.4 mL/min, the injection volume was 3 μL and the column temperature was 45°C. The mass spectrum signals were adopted by the positive and negative ion scanning mode. The electrospray capillary voltage, the injection voltage and the collision voltage were 1 kV, 40 V, and 6eV. The temperatures of the ion source and desolvation were 120°C and 500°C. The carrier gas flow rate was 900 L/h, with the scanning area ranging from 50 to 1000 m/z, the scanning time being 0.1 s and the interval time being 0.02 s.

The quality control (QC) sample was mixed by all the detected samples with equal volume. The volume of every QC sample and each of the samples was the same, with the same treatment and detection methods. During UPLC-MS analysis, every eight analyzed samples will be added one QC sample to guarantee the repeatability of the analytical method.

### Metabolites Identification in *Polyporus umbellatus*

The raw data were first identified based on the mass and then tested using the secondary spectrum analysis and metabolomic processing software Progenesis QI (Waters Corporation, Milford, MA, United States) to filter the baseline, identify and integrate the peaks, correct the retention time and perform peak alignment and normalization. According to the spectrograms of the reference standards, the scores of the sample spectrum were rated and the identification confidence levels in high resolution mass spectrometric analysis were conducted (Schymanski et al., 2014). The metabolites were identified by matching high resolution mass to compounds in databases such as Metlin database^1^, human metabolome database (HMDB^2^) and self-built Chinese herbal database, combined with fragmental score or theoretical fragmentation score more than 45 and the metabolites originated from fungi were selected. The mass tolerance between the exact mass and the measured m/z values was less than10 ppm (Yi et al., 2020).

### Differential Metabolites Screening and Identification

#### Multivariate Statistical Analysis

Multivariate statistical analysis was carried out. The normalized data matrix was input to SIMCA-P + 14.0 software package (Umetrics, Umeå, Sweden). First, the data was subjected to unsupervised principal component analysis (PCA) to evaluate the population distribution among all the samples and the stability all through the analyzed process. Subsequently, supervised (orthogonal) partial least square analysis [(O)PLS-DA] was used to distinguish the overall differences of the metabolism outline and find different metabolites between groups. During (O)PLS-DA analysis, variable important in projection (VIP) >1 was considered to be difference variables. To prevent over-fitting of the model, 7 cycles of cross-validation and 200 times of response sequencing test were used to assess the quality of the model.

Differential metabolites between groups were screened using combined OPLS-DA (multi-dimensional analysis) and Student *T*-test (one-dimensional analysis), based on the variable important in projection (VIP) >1 and *P* < 0.05. Total ion current of all samples including QC sample were visualized. Differential metabolites were identified by means of database searching. The public databases can be obtained on the websites of^3^^,4^ (see text footnote 2) and there are also self-built databases such as Chinese herbal database to be used.

#### Identification of Ergosterol Synthesis-Related Genes and Their Expression Profiles in *Polyporus umbellatus* After *Armillaria* sp. Infection

As *A. mellea* refernce genomic information has been published online, in our previous study, using Bowtie Program (0.12.8) (Langmead et al., 2009), the *A. mellea*’s reads in the QR group has been removed by means of mapping all the reads according to *A. mellea* reference genomic data (Liu et al., 2015). Based on the transcriptome analysis of *P. umbellatus* sclerotia infected by *A. mellea*, DEGs in the QR group related to steroids especially ergosterol synthesis were analyzed, combined with that of the control group.

#### Validation of the Differentially Expressed Genes Related to Ergosterol Synthesis Expression Using qRT-PCR

The primers (Supplementary Table 1) were designed with Primer 3 input (v. 0.4.0) from^5^ (Koressaar and Remm, 2007; Untergasser et al., 2012) and synthesized by Genewiz Company of China. The *ß-tubulin* gene with the accession number of EU442274 was selected as a reference control in *P. umbellatus* (Liu et al., 2015). According to the transcriptome analysis, the DEGs of *comp16933_c0*, *comp34626_c0* and *comp32446_c0*, respectively, encoding ERG6, ERG2 and ERG5 were selected for validating their expression patterns in the QR and the control groups. The PCR-amplification, the qRT-PCR protocol were all conducted using Real-time fluorescence quantitative PCR instrument (Roche, LightCycler 480), according to a previous study (Xing et al., 2013, 2020).

#### Verification of the Level Changes of Ergosterol, Polyporusterone A and Polyporusterone B Between the QR and Control Group

The reference substances of ergosterol, polyporusterone A and polyporusterone B were bought from Chengdu Pulis Biological Technology Co., Ltd. The fresh *P. umbellatus* sclerotia were washed clean by running water and then dried. After the samples of the QR and the control groups were divided, respectively, *P. umbellatus* sclerotia in both groups were smashed and filtered with 40-mesh-sieve. Then the reference substances of ergosterol, polyporusterone A and B with 1.76 mg, respectively, were measured and dissolved in methanol solution in a volumetric flask with the accurate volume of 2 ml. The concentration of the reference solution was set to 0.88 mg/ml. After ultrasonic treatment to accelerate the dissolution, different volumes of the single reference substance were extracted and mixed, then 180 μL ergosterol and 450 μL polyporusterone A or B were added in a 1 mL-volumetric-flask, which was the mixed reference substance solution for quantitative analysis. Each sample 1.0 g were weighed and soaked it in 95% ethanol solution of 20 times volume for 12 h, after ultrasonic extraction for 1 h, the samples were filtered. Subsequently, the filtrate was concentrated, and the ethanol solvent was volatilized to be completely dried, the residue was in constant volume to 1 mL in a volumetric flask with methanol solution. The HPLC detection system includes Waters 2996 detector, Waters 2707 sampler and Waters 600 four-element pump. The determination was performed on a Bridge RP18 Column (250 mm × 4.6 mm, 5 μm). The flow rate was 1.0 mL/min, and the column temperature was 30°C. The detection wavelength was 247.4 nm for polyporusterone A or B and 283 nm for ergosterol. Before injection, the reference substance and the sample solution were filtered with the organic phase filtration membrane (0.22 μm). The Mobile phase for analysis was set as follows: A phase: 10% acetonitrile, B phase: acetonitrile, C phase: methanol. The detailed chromatographic conditions were listed in Supplementary Table 2.

### Statistical Analysis

The data for the content detection were analysed using SPSS 11.0 (SPSS, Chicago, IL, United States), with a *t*-test method. All the data were expressed as the means ± SD from at least three independent experiments. *P*-value < 0.05 was regarded as significant difference.

## Results

### Differentially Expressed Metabolites of Untargeted UPLC-MS Metabolomic Analysis

In total, 258 metabolites were identified from database Metlin, human metabolome database (HMDB) and Chinese herbal related database with fragmentation score or theoretical fragmentation scores more than 45, including fatty acid (such as Alpha-Linolenic acid, 13-L-Hydroperoxylinoleic acid, Tetradecanedioic acid, 12-hydroxyheptadecanoic acid, et al.), carbohydrate (Sucrose, Galactose-beta-1,4-xylose, L-arabinose and Stachyose, et al.) and steroids (Supplementary Table 2). Because the QR part did not contain *A. mellea*, in the 258 identified metabolites, no characteristic metabolites of *A. mellea* such as armillaridin or melleolide were detected (Wang et al., 2016).

**FIGURE 2:**
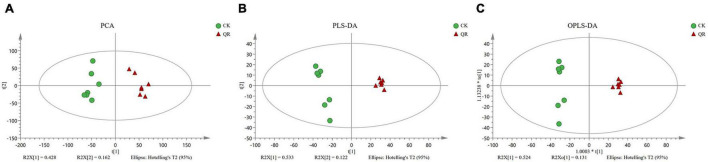
Multivariate analysis of UPLC-MS data. **(A)** PCA; **(B)** PLS-DA; **(C)** OPLS-DA. The green circles and the red triangles, respectively, stand for seven biological replicates in the QR and the control groups.

Principal component analysis was used to demonstrate the trends, outliers and the general clustering (Wen et al., 2019). In the present study, PCA showed that the significant difference in the metabolic profilings between the QR group and the control group (Figure 2). Besides, OPLS-DA, PLS-DA was also used to analyze the data. SPSS 11.0 (SPSS Inc., Chicago, IL) was used to perform the statistical analysis.

118 DEMs between the QR and the control groups were obtained based on VIP > 1 and *P* < 0.05 and of these metabolites, there were steroids that we were interested in, containing steroids including Ergosterol, Polyporusterone A, B, and G, (22E, 24x)-Ergosta-4,6,8,22-tetraen-3-one, etc., (Table 1).

**TABLE 1:** Characteristics of the DEMs by UPLC-MS.

ID	Metabolite	m/z	Retention time	Mode	Adducts	Formula	Mass error	Comp library ID	CAS ID
Pos_147	(22 E, 24×) Ergosta- 4,6,8,22- tetraen-3 - one	393.3145	10.0354	Pos	M+H, M+Na, 2M+Na	C28H40O	–1.769	HMDB0030898	194721-75-0
Pos_612	3,5-Dihydroxyergosta- 7,22 - di en- 6- one	451.3179	9.2830	Pos	M+H, M+Na	C28H44O3	–9.261	HMDB0031953	–
Pos_4213	Polyporusterone G	461.3257	5.9819	Pos	M+H	C28H44O5	–0.897	HMDB0038501	141360-94-3
Pos_4217	Polyporusterone B	477.3211	5.9960	Pos	M+H	C28H44O6	0.005	HMDB0038496	141360-89-6
Pos_4218	Polyporusterone A	479.3365	5.9819	Pos	M+H, M+Na, M+H-2H20	C28H46O6	–0.428	HMDB0038495	141360-88-5
Pos_4262	Ergosterol	379.3357	8.9688	Pos	M+H-H20, M+H	C28H44O	–0.508	LMST01030093;HMDB0000878	57-87-4
Pos_528	(3beta,22E, 24R) -Ergosta-4,6,8(14),22- tetraen- 3- o1	377.3198	10.4209	Pos	M+H-H20, M+H	C28H42O	–1.204	HMDB0041050	104729-39-7
pos_3082	(2beta,3 alpha,9 alpha,24R) -Ergosta-7,22 - diene- 2,3,9 -triol	395.3282	8.24178	Pos	M+H-2H20	C28H46O3	–6.049	HMDB0037914	–
Neg_2933	(3beta,5 alpha,6 alpha,9 alpha,22E, 24R) -Ergosta-7,22- diene-3,5,6,9-tetrol	491.3364	7.9946	Neg	M+FA-H	C28H46O4	–3.127	HMDB0032123	211486-15-6
Neg_1322	(3beta,5 alpha,9 alpha,22E, 24R) -5,9- Epidioxy-3-hydroxyergosta-7,22-dien-6-one - hy dr ox yer gosta	487.3045	7.9946	Neg	M+FA-H	C28H42O4	–4.545	HMDB0032666	211486-16-7

### Ergosterol Biosynthetic Pathway

**FIGURE 3:**
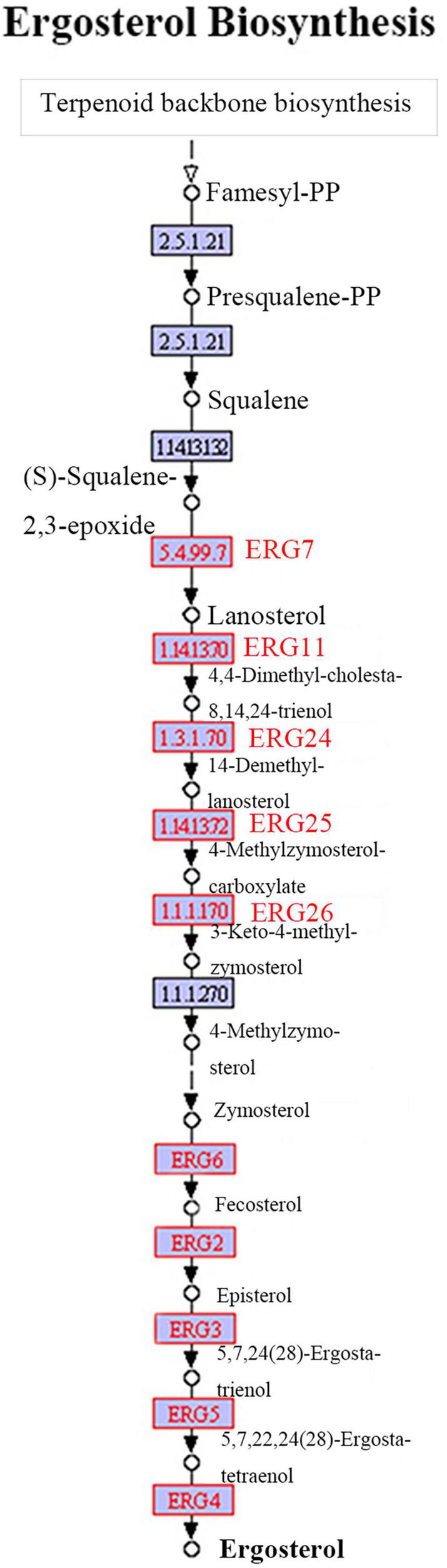
Ergosterol biosynthetic pathway. The ergosterol biosynthetic pathway from Terpenoid backbone to ergosterol has been shown. The words in red represent DEGs up-regulated in the QR group, compared to that of the control group.

The ergosterol biosynthesis information from the transcriptome of *P. umbellatus* infected by *A. mellea* can be obtained from^6^ and the Illumina sequencing data can be obtained from the SRA database (SRP058382). Compared to that of the control group, differentially expressed genes encoding ERG 7, ERG 11, ERG 24, ERG 25, ERG 26, ERG 6, ERG 2, ERG 3, ERG 5, and ERG 4 may serve as the key enzymes in the QR group and were upregulated during the ergosterol biosynthesis (Figure 3). All the DEGs related to the ergosterol biosynthesis were listed in Supplementary Table 4.

### Validation of the Expression of the Differentially Expressed Genes Related to Ergosterol Synthesis Expression

**FIGURE 4:**
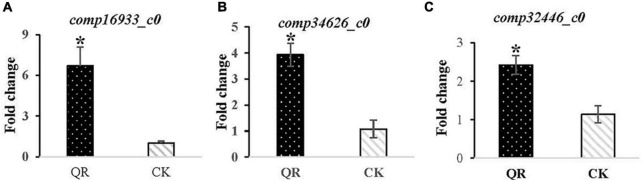
Validation of the DEGs related to ergosterol synthesis expression using qRT-PCR. The ERG6 encoded by *comp16933_c0*, the ERG2 encoded by *comp34626_c0* and the ERG5 encoded by *comp32446_c0* all presented up-regulated 6.71 **(A)**, 3.93 **(B)** and 2.42 **(C)** folds, respectively, in the QR group. * stands for *P* < 0.05, in comparison to that of the control group.

The expression of the three DEGs related to ergosterol synthesis were performed using qRT-PCR to validate the facticity of the transcriptomic data. The result indicated that the *comp16933_c0*, *comp34626_c0* and *comp32446_c0* encoding ERG6, ERG2 and ERG5 were all up-regulated 6.71 (Figure 4A), 3.93 (Figure 4B) and 2.42 (Figure 4C) folds, respectively, in the QR group, compared to that of the control group.

### Prediction of Steroids Transformation From Ergosterol to Polyporusterone A in *Polyporus umbellatus*

**FIGURE 5:**
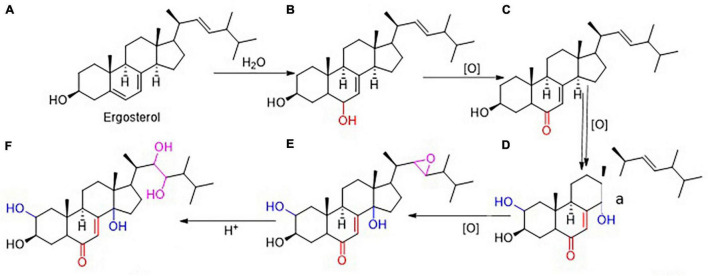
Prediction of Steroids in *P. umbellatus* transformation. Based on the chemical reactions and series of oxidized reactions, it was deduced that Ergosterol **(A)** gradually could be converted into Polyporusterone A **(F)**. During the transition process, **(B–E)** respectively represented 3,6-dihydroxyergosta-7,22-diene, 3-hydroxyergosta-7,22-diene-6-one, 2,3,14-trihydroxyergosta-7,22-diene-6-one and 2,3-dihydroxyergosta-7-en-6-one-22,23-epoxy.

Based on the chemical reactions, Ergosterol (A) can be converted into the hydroxylation product at the sixth position (B), subsequently the product will be oxidized into α,β-unsaturate ketone (C). This product will turn into 2,14-dihydroxylation product (D) by oxidase. Then the double bond of the carbon in the 22th position will be catalyzed by oxidase to form new unstable and transitional product with three oxygen bridges (E). After that, the compound five converted into Polyporusterone A (F) with the participation of H^+^ (Figure 5).

### The Content of Ergosterol, Polyporusterone A and B in the QR and the Control Groups

Based on our previous study, ergosterol was isolated and identified from *P. umbellatus* sclerotia (Zhou and Guo, 2009) and the retention time of ergosterol, polyporusterone A and B in *P. umbellatus* sclerotia was in accordance with that of the standard substances (Supplementary Figure 1). After cultivation with *A. mellea* for 1 year and a half in the field, the ergosterol, polyporusterone A and B in the QR group of *P. umbellatus* was increased 32.2, 75.0, and 20.0%, compared to that of the control group (Table 2). This showed the validation of the metabolomic data. Conversely, the result indicates that *A. mellea* drives chemical compound changes in *P. umbellatus* sclerotia.

**TABLE 2:** The content of ergosterol, polyporusterone A and B.

Groups	Ergosterol (μg/g)	Polyporusterone A (μg/g)	Polyporusterone B (ug/g)
QR	110.08±8.31*	7.10±0.72*	3.00±0.31*
CK	83.80±8.43	4.050±0.41	2.50±0.23

Data was represented by means ± SD from three independent experiments, with three replicates in each group, * represented P < 0.05 (compared to the control group).

## Discussion

*Armillaria mellea* is a type of saprophytic, parasitic and facultative fungus. *P. umbellatus* sclerotia can be generated only after it is co-cultured with *A. mellea*. In addition, *A. mellea* can establish a symbiotic relationship with *Gastrodia elata* (Orchidaceae), a well-known traditional Chinese medicinal plant, in which the gastrodin is widely used in China (Qiao et al., 2021). *A. mellea* is the nutrient supplier of *P. umbellatus* or *G. elata* (Guo and Xu, 1990, 1992), although the effect of *A. mellea* on *P. umbellatus* is not limited to this function (Baumgartner et al., 2011). The results of this study have demonstrated that *A. mellea* can also drive the changes of the chemical components of *P. umbellatus* sclerotia such as fatty acids, amino acids, sugars and steroids including ergosterol, Polyporusterone A and B (Table 1 and Supplementary Table 2).

In fungi, sterol biosynthesis is highly conserved (Ferreira et al., 2005). As a vital component of the fungal cell membranes, the ergosterol plays essential roles in regulating the permeability, fluidity and integrity of the membrane in fungi (Jordá and Puig, 2020). Ergosterol serves as the precursor of vitamin D2 and other steroid hormones. In *Saccharomyces cerevisiae*, squalene synthase (Erg 9) catalyzes farnesyl-PP to produce squalene and lanosterol is produced from squalene by Erg7 (lanosterol synthase). Subsequently, zymosterol changed from lanosterol by Erg11, Erg 25, Erg26, and Erg27 (Liu et al., 2019). Then, zymosterol is transformed to fecosterol by Erg6 and the latter is converted to episterol catalyzed by Erg2. After a series of reactions catalyzed by Erg3, 5, and 4, ergosterol is produced. In *P. umbellatus*, many DEGs such as ERG7, ERG11, ERG24, ERG25, ERG26, as well as ERG6, EGR2, ERG3, ERG5, and ERG4 were all up-regulated expressed in the QR group, compared to that of the control group. All the selected three DEGs of ERG6, ERG2, and ERG5 in the later part of the biosynthesis of ergosterol indicated that the tendency of the expression patterns at the mRNA level by qRT-PCR was in accordance with that of the RNA-Seq information. During ergosterol biosynthesis, the ERG2 is an enzyme catalyzing the transformation of delta 8 double bond to the delta seven position (Ashman et al., 1991). It has been reported that ERG6 and ERG2 serve as important targets in conferring reduced susceptibility to amphofericin B in *Candida glabrata* (Ahmad et al., 2019). ERG5 belongs to the cytochrome P450 and participates in ergocalciferol biosynthesis (Jordá and Puig, 2020). In *Neurospora crassa* and *Fusarium verticillioides*, deletion of *ERG5* leads to hypersensitive to antifungal azoles (Sun et al., 2013).

Polyporusterone A is a secondary metabolite of *P. umbellatus* sclerotia (Bandara et al., 2015). *A. mellea* can increase the content of Polyporusterone A in sclerotia after symbiotic with *A. mellea* (Table 2), indicating that there is probably one or more biological pathway for *A. mellea* to regulate the content of the components in *P. umbellatus* sclerotia. Based on the combined analysis of metabolomic and transcriptomic analysis, this study revealed the possible pathway of *A. mellea* regulating Polyporusterone A production in sclerotia. Up to now, there are few reports on the regulation of the chemical components driven by *A. mellea*, which is inconsistent with the characteristics of *A. mellea* being the largest and highly saprophytic (forest scavenger) in the world (Smith et al., 1992; Anderson and Catona, 2014). After the symbiotic relationship between *A. mellea* and *G. elata* was established, *A. mellea* can regulate the content of gastrodin (Tsai et al., 2016), a secondary metabolite of *G. elata*, which may be related to the defense of *A. mellea*. The role of steroids in the symbiotic relationship between *A. mellea* and *P. umbellatus* needs to be further studied, and this study provides new insight for the follow-up in-depth study of the interaction mechanism between *A. mellea* and *P. umbellatus*.

## Data Availability Statement

The datasets presented in this study can be found in online repositories. The names of the repository/repositories and accession number(s) can be found in the article/Supplementary Material.

## Author Contributions

S-XG and JC: experimental design and manuscript review. Y-MX: performing the experiments, manuscript writing and review. BL: part of the manuscript writing and review. BL and LL: performing part of the experiments. YL: data analysis and manuscript review. S-XY and S-CY: cultivating *P. umbellatus* and sample collection. All authors have read and approved the manuscript.

## Conflict of Interest

S-XY and S-CY were employed by Qinzheng Zhuling Development Co., Ltd., of Liuba, Shaan’xi, China. The remaining authors declare that the research was conducted in the absence of any commercial or financial relationships that could be construed as a potential conflict of interest.

## Publisher’s Note

All claims expressed in this article are solely those of the authors and do not necessarily represent those of their affiliated organizations, or those of the publisher, the editors and the reviewers. Any product that may be evaluated in this article, or claim that may be made by its manufacturer, is not guaranteed or endorsed by the publisher.

## Footnotes

1
https://ngdc.cncb.ac.cn/databasecommons/

2
https://hmdb.ca/

3
http://metlin.scripps.edu

4
http://www.lipidmaps.org/

5
https://bioinfo.ut.ee/primer3-0.4.0/

6
https://www.genome.jp/pathway/ko00100
