# Supplementary material for: Armillaria mellea Symbiosis Drives Metabolomic and Transcriptomic Changes in Polyporus umbellatus Sclerotia
Source: Front Microbiol. 2022 Feb 3;12:792530. doi: 10.3389/fmicb.2021.792530 (PMC8851056; doi:10.3389/fmicb.2021.792530)
Supplement: Supplementary file 1 [file Data_Sheet_1.docx]

Supplementary Material

Supplementary Table 1. The Primer sequences used in qRT-PCR method.

| DEGs No. | Forward | Reverse |
| --- | --- | --- |
| *comp32446_c0* | CGCAATGTCCTGCTGTATCT | CTCCTGTGGTGGAAGCATATC |
| *comp16993_c0* | CAGGATCAAGCCAGTGATAGG | GTTAGCAGTGGTCGGGATAAG |
| *comp34626_c0* | CCGCGAACAAGTCAAAGTAAAC | TGAATAGGTCCGGTCGAGATAG |
| *ß-tubulin* | CCTTCCTTGGCAACTCGACA | TCGTCCATACCCTCCTGTGT |

**Supplementary table 3. The chromatographic conditions of detection of ergosterol, polyporusterone A and B**

|  | Time (min) | Flow | % A | % B | % C | Curve |
| --- | --- | --- | --- | --- | --- | --- |
| 1 | 0 | 1.00 | 78.0 | 22.0 | 0.0 | 6 |
| 2 | 24.00 | 1.00 | 78.0 | 22.0 | 0.0 | 6 |
| 3 | 24.10 | 1.00 | 0.0 | 0.0 | 100.0 | 6 |
| 4 | 44.00 | 1.00 | 0.0 | 0.0 | 100.0 | 6 |
| 5 | 44.50 | 1.00 | 78.0 | 22.0 | 0.0 | 6 |
| 6 | 50.00 | 1.00 | 78.0 | 22.0 | 0.0 | 6 |

**Supplementary Table 4. DEGs related to the ergosterol biosynthesis**

| Gene NO. | Log_2_Fold change | Adjusted *p*-value | Up or down regulation | Enzyme Annotation |
| --- | --- | --- | --- | --- |
| *comp25003_c0* | Inf | 0.00073437 | up | Lanosterol synthase |
| *comp18183_c0* | Inf | 0.000000000000554 | up | Lanosterol 14-alpha demethylase |
| *comp24208_c0* | Inf | 0.000000000541 | up | Delta14-sterol reductase (ERG24) |
| *comp10804_c0* | Inf | 0.0000000000792 | up | Methylsterol monooxygenase |
| *comp13390_c0* | Inf | 0.036547 | up | Sterol-4alpha-carboxylate 3-dehydrogenase (decarboxylating) |
| *comp31560_c0* | Inf | 0.0000149 | up |  |
| *comp16993_c0* | 1.2861 | 0.045057 | up | sterol 24-C-methyltransferase (ERG6) |
| *comp26622_c0* | Inf | 9.21E-21 | up |  |
| *comp34626_c0* | 1.8143 | 0.00000995 | up | C-8 sterol isomerase (ERG2) |
| *comp20073_c0* | Inf | 0.00030883 | up |  |
| *comp17913_c0* | 1.76 | 0.00021605 | up | Delta7-sterol 5-desaturase (ERG3) |
| *comp27743_c0* | Inf | 0.00012043 | up |  |
| *comp20500_c0* | Inf | 0.035968 | up |  |
| *comp24287_c0* | Inf | 0.0028388 | up | Delta24(24(1))-sterol reductase |
| *Comp31596_c0* | Inf | 0.0028388 | up |  |
| *comp31087_c0* | Inf | 0.00000346 | up | Sterol 22-desaturase (ERG5) |
| *comp23006_c0* | Inf | 0.00000000136 | up |  |
| *comp32446_c0* | 2.2947 | 1.82E-38 | up |  |
| *comp24975_c0* | Inf | 0.045057 | up |  |

**
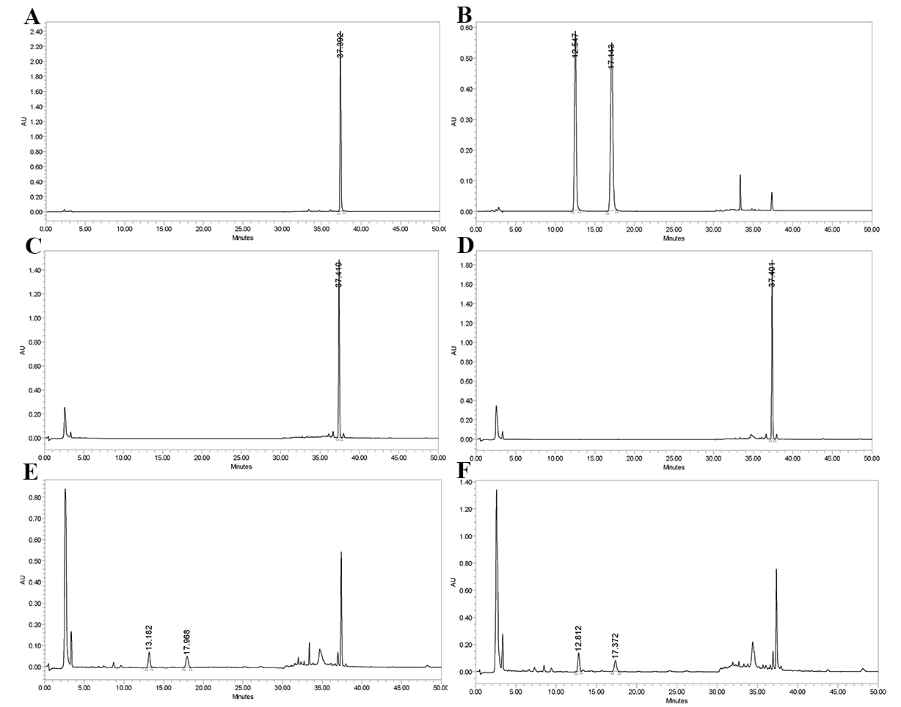
**

**Figure captions**

**Supplementary figure 1. Chromatograms of the reference substances and the samples.**

A stands for the chromatogram of the reference substances of ergosterol.

B represents the chromatograms polyporusterone A and polyporusterone B.

C and D respectively stand for the chromatograms of the ergosterol in the control group and the QR group.

E and F respectively represent the chromatograms of the polyporusterone A and polyporusterone B in the control group and the QR group.
